# The GSTM2 C-Terminal Domain Depresses Contractility and Ca^2+^ Transients in Neonatal Rat Ventricular Cardiomyocytes

**DOI:** 10.1371/journal.pone.0162415

**Published:** 2016-09-09

**Authors:** Ruwani P. Hewawasam, Dan Liu, Marco G. Casarotto, Philip G. Board, Angela F. Dulhunty

**Affiliations:** John Curtin School of Medical Research, The Australian National University, GPO Box 334, Canberra City, ACT 2600, Australia; University of Debrecen, HUNGARY

## Abstract

The cardiac ryanodine receptor (RyR2) is an intracellular ion channel that regulates Ca^2+^ release from the sarcoplasmic reticulum (SR) during excitation–contraction coupling in the heart. The glutathione transferases (GSTs) are a family of phase II detoxification enzymes with additional functions including the selective inhibition of RyR2, with therapeutic implications. The C-terminal half of GSTM2 (GSTM2C) is essential for RyR2 inhibition, and mutations F157A and Y160A within GSTM2C prevent the inhibitory action. Our objective in this investigation was to determine whether GSTM2C can enter cultured rat neonatal ventricular cardiomyocytes and influence contractility. We show that oregon green-tagged GSTM2C (at 1 μM) is internalized into the myocytes and it reduces spontaneous contraction frequency and myocyte shortening. Field stimulation of myocytes evoked contraction in the same percentage of myocytes treated either with media alone or media plus 15 μM GSTM2C. Myocyte shortening during contraction was significantly reduced by exposure to 15 μM GSTM2C, but not 5 and 10 μM GSTM2C and was unaffected by exposure to 15 μM of the mutants Y160A or F157A. The amplitude of the Ca^2+^ transient in the 15 μM GSTM2C - treated myocytes was significantly decreased, the rise time was significantly longer and the decay time was significantly shorter than in control myocytes. The Ca^2+^ transient was not altered by exposure to Y160A or F157A. The results are consistent with GSTM2C entering the myocytes and inhibiting RyR2, in a manner that indicates a possible therapeutic potential for treatment of arrhythmia in the neonatal heart.

## Introduction

The glutathione transferases (EC 2.5.1.18) (GSTs) are a major family of phase II detoxification enzymes that conjugate the tripeptide glutathione (GSH) to a wide range of endogenous and exogenous toxins [1]. GSTs also have a range of other significant functions and are implicated in the modulation of cell signaling kinases such as JUNK and ASK-1 [2–4], the synthesis of steroid hormones [5], the catabolism of tyrosine [6, 7] and in the activation of interleukin 1β [8]. We discovered that members of GST structural family modulate ryanodine receptor (RyR) Ca^2+^ release channels isolated from cardiac and skeletal muscle [9]. GSTM2 is specifically expressed in human skeletal and cardiac muscle [10] and was identified as an inhibitor of cardiac ryanodine receptor (RyR2), but not skeletal ryanodine receptor (RyR1), channels [11, 12]. RyRs are a class of ligand-gated cation channels that are embedded in the membrane of intracellular Ca^2+^ stores within endoplasmic reticulum (ER) in smooth muscle and non-muscle cells and sarcoplasmic reticulum (SR) in striated muscle fibers [13, 14]. The normal physiological function of GSTM2, in addition to its GSH conjugation, appears to be an anti-arrhythmic stabilization of RyR2 activity [11, 12] which can be overridden by pro-arrhythmic factors in cardiac disorders. Therefore the selective inhibition of RyR2 by the C-terminal half of GSTM2 (GSTM2C) has significant clinical potential in the treatment of inherited and acquired RyR Ca^2+^-handling based arrhythmia where RyR2 channels are abnormally active, inducing arrhythmias and reducing Ca^2+^ store filling during diastole and contractility.

**Fig 1:**
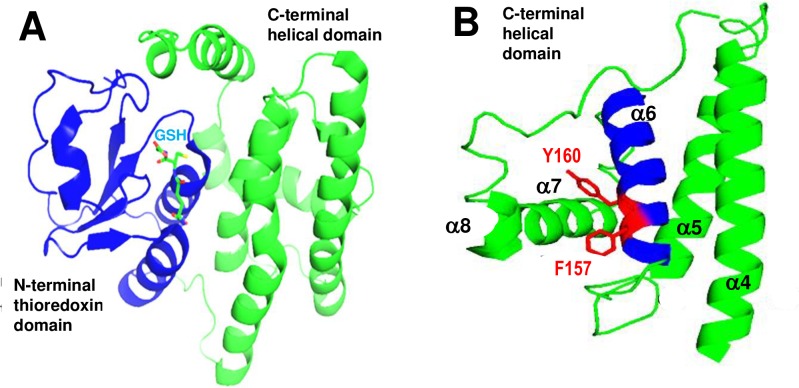
Structure of GSTM2 and GSTM2C. **A**: GSTM2 with the N-terminal thioredoxin domain shown in blue and the C-terminal helical domain in green. The binding site for reduced glutathione is indicated in light blue (structure from PDB entry 1XW5 [19]). **B**: The C-terminal domain, GSTM2C, containing α helices 4–8 labelled α4 – α8. Helix α6 is highlighted in blue and residues F157 and Y160 located in helix 6 shown in red with predicted stabilizing interactions with helix 7 [20]. The GSTM2C structure is predicted from the complete crystal structure (PDB entry 1XW5 [19]).

We have shown that GSTM2 binds to RyR2 through its C-terminal domain and that this α-helical domain inhibits RyR2 function, but has no effect on skeletal muscle RyR1 Ca^2+^ channels [11]. Helix 6 is a core element of GSTM2C that is essential for RyR2 inhibition [12]. Helix 6 has also been shown to play a key role in global folding of the cytosolic GST family [15]. Specific mutations in helix 6 (Fig 1) showed that two residues, F157A and Y160A prevent the C-terminal half of the protein from exerting its normal inhibitory action on RyR2 channels [11]. Furthermore, we find that the selective inhibition of RyR2 by GSTM2C is due to its interaction with amino acids within the divergent region 3 (D3 region) of cardiac ryanodine receptor [16]. We have also shown that GSTM2C can enter cells and carry cargo [17] and is taken up by adult mouse cardiomyocytes, altering Ca^2+^ signaling and contraction in a manner consistent with a direct action on RyR2 [18].

A remaining question is whether GSTM2C has a similar action on neonatal cardiomyocytes where Ca^2+^ signaling during contraction depends on a different combination of ion channels, including a contribution of Ca^2+^ from inositol 1,4,5-trisphosphate (IP3) sensitive stores to contraction [21]. In contrast to adult myocytes, it has been reported that the changes in the excitation-contraction coupling occurring during a 7 day postnatal period in rat cardiomyocytes are essentially the same in primary culture and in the postnatal period in vivo [22]. Therefore the objective of the present investigation was to determine whether GSTM2C enters cultured neonatal ventricular cardiomyocytes and to assess the effect of GSTM2C and its mutants F157A, Y160A on the contractility and Ca^2+^ transients in these cells, to evaluate the generality of the use of GSTM2C to suppress RyR2 activity at different stages of cellular development. The results showed that GSTM2C can indeed enter these cultured neonatal myocytes and reduces spontaneous and externally electrically triggered contractility and Ca^2+^ transients.

## Methods

### Reagents

The plasmids for the expression of recombinant GSTM2C and its mutants F157A and Y160A were constructed as described in our previous paper [12]. All other chemicals were purchased from Sigma–Aldrich Pty. Ltd (PO Box 970, Castle Hill, NSW 1765, Australia.

### Expression and purification of recombinant GSTM2C

Recombinant GSTM2C was expressed in E. coli from a pHUE vector and purified by Ni-agarose affinity chromatography as previously described [11]. The poly His-ubiquitin tag used for the affinity chromatography was subsequently cleaved by digestion with a catalytic fragment of the deubiquitylating enzyme mouse Usp 2 [23]. This strategy provides recombinant GSTM2C without any additional residues [12]. The numbering of residues mutated in the C-terminal domain is based on the whole GSTM2 protein.

### Isolation of neonatal ventricular cardiomyocytes from rats

The isolation and culture of cardiomyocytes was conducted as described in [24]. All experiments were specifically approved by the Animal Experimentation Ethics Committee (AEEC) of the Australian National University, and carried out according to the guidelines of the AEEC in compliance with the Australian Code of Practice for the Care and Use of Animals for Scientific Purposes. Neonatal Wistar rats (1–2 days old) obtained from the ANU Bioscience Services were sacrificed by decapitation with sharp surgical scissors. The dissociated ventricular cells were transferred to collagen coated plates and cultured in Complete Dulbecco's modified Eagle's medium (CDMEM). Fetal calf serum was omitted from the culture media to avoid massive fibroblast contamination of the primary cultures. All experiments were performed on day 4 of culture.

### Immunostaining with anti-α-actinin

Cells were fixed with 4% paraformaldehyde for 20 min, permeabilized with 0.1% Triton-X-100 in PBS for 10 min and incubated for 1 h with monoclonal anti α-actinin (sarcomeric) primary antibody at 1:200 dilution. Secondary antibody, Alexafluor 568 goat anti mouse IgG (excitation/emission maxima ~578/603 nm), was added at 1:100 dilution and observed in Leica SP5 confocal microscope (Leica, Mannheim, Germany). The immunostaining allowed distinction of cardiomyocytes from fibroblasts and then allowed confirmation that the cardiomyocytes were able to accumulate GSTM2C.

### GSTM2C uptake into cardiomyocytes

GSTM2C was fluorescently labelled with Oregon Green (GSTM2C-OG) [17]. Cardiomyocytes were incubated with 1 μM GSTM2C-OG for 24 h to allow maximal uptake as some material may have been lost during Triton-X-100 treatment in experiments requiring subsequent immunostaining. In this experiment cells mounted on coverslips were thoroughly washed and observed under Leica SP5 confocal microscope and a series of images were taken through the Z-plane. The time course of uptake of GSTM2C into spontaneously contracting cardiomyocytes was also determined. Cells mounted on coverslips were treated with 1 μM GSTM2C-OG, and images were taken every 20 min up to 3 h. Oregon Green Fluorescence emission was captured at 514 nm using an argon 488 nm laser line.

### Spontaneous contraction

Cardiomyocytes were placed on a heated stage maintained at 37°C with CO_2_/air (5%/95%) flowing over the cells. Images of contracting myocytes were recorded using a JVC video camera attached to a Nikon TE2000-U microscope. Cells were kept on the stage without changing the field of view. Then the CDMEM was exchanged with fresh CDMEM containing 15 μM WT or mutant GSTM2C and images of contractions again recorded after 2 h. Images of relaxed and maximally shortened myocytes were selected. A line was drawn along the axis of contraction within the cell between landmark points that were visible before and after incubation. The maximum and minimum lengths respectively measured with Image pro plus 6.2 software.

### Field stimulated contraction

Cells were stimulated via a pair of parallel Pt electrodes located 2 mm apart on either side of the coverslip with 2 ms, ~3 V pulses, delivered in a train of 5 pulses at 1 Hz. The polarity of the stimulus was reversed every fourth train to avoid buildup of electrolyte by-products. To optimize the pulse parameters, the voltage was slowly increased until threshold contraction was observed, then the voltage adjusted to a level that was 20% higher than the threshold. Control contractions were recorded at the beginning of the experiment and then the cells were treated for 2 h with 5, 10 or 15 μM GSTM2C, or mutants, while on the stage without changing the field of view and without stimulation. After 2 h the same cells were stimulated and contraction recorded. The most contracted and relaxed images were selected and analysed in the same way as spontaneous contraction (2.0.6).

### Ca^2+^ transients

The cardiomyocytes were treated with either vehicle alone (control) or vehicle plus GSTM2C constructs at a final concentration of 15 μM and incubated for 2 h. The cells were then loaded with the fluo-4 AM Ca^2+^ indicator and imaged using a Leica SP5 confocal microscope in line scan (*x-t*) mode. Fluo 4 AM was excited using the 488 nm Argon laser and the line scan was positioned parallel to the longitudinal axis of the cell. Cells were paced at 1 Hz using a 2 ms pulse with a voltage approximately 20% above the contraction threshold. Leica Application Suite Advanced Fluorescence software was used for data acquisition; to correct line scans for background fluorescence and to calculate relative fluorescence (ΔF/F0). Axograph X software (Axograph, Berkely, USA) was used to obtain values for the peak amplitude, rise time from 10% to 90% of the peak and decay time from the peak to 50% of the peak of the ΔF/F0 transients.

### Statistics

Average data are given as mean ± SEM. Statistical significance was evaluated using paired or unpaired Student’s t-test as appropriate. A P value of <0.05 was considered significant.

## Results

### Identification of cardiomyocytes and uptake of GSTMC-OG

**Fig 2:**
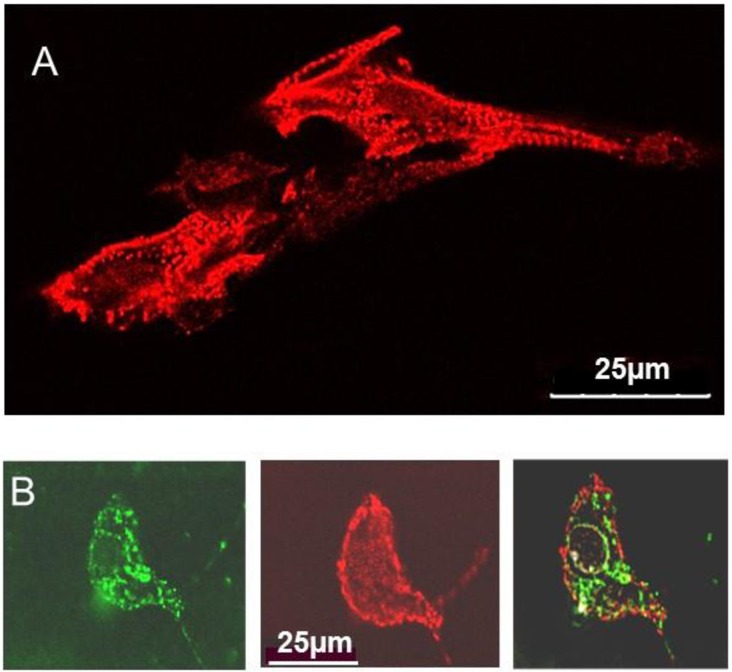
Cultured myocytes, identified by α-actinin staining, accumulate Oregon Green conjugated GSTM2C. **A:** Longitudinal view of fixed cultured neonatal cardiomyocytes showing the striations of Z-lines labelled with α-actinin when regions of the myocytes were oriented with Z-lines at right angles to the beam. **B:** A transversely sectioned cardiomyocyte that had been exposed to GSTM2C-OG for 24 h, then fixed and immunostained with α-actinin antibody. Left–strong accumulation of GSTM2C-OG within the cytoplasm in a typical punctate distribution. Middle– α-actinin antibody staining of cross-sectioned Z-lines. Right–superimposed GSTM2C-OG and α-actinin stained images showing cross-sectioned Z-lines within the GSTM2C-OG stained cytoplasm.

In initial experiments, the neonatal myocytes were immuno-stained with anti α-actinin to distinguish cardiomyocytes from fibroblasts [25]. As expected the anti-α-actinin antibody stained Z lines, and thus revealed the typical striations in appropriately oriented regions of the myocytes (Fig 2A) [26]. Fig 2B (left) shows that a myocyte exposed GSTM2C-OG for 24 h had accumulated the tagged protein with a characteristic punctuate green fluorescence pattern. The same myocyte showed anti-α-actinin staining (Fig 2B center) and there was a partial overlap of GSTM2C and α-actinin (Fig 2C right). Therefore GSTM2C does enter neonatal cardiomyocytes.

It is notable that there were few fibroblasts in the preparations as precautions were taken to minimise fibroblast numbers (Methods). However there appeared to be little GSTM2C in the fibroblasts that were there, with the GSTM2C concentrations and loading times that we used. Our previous data suggests that all cells take up GSTs, but in very different amounts, likely due to different rates of uptake [17]. Therefore it is likely that fibroblasts accumulate GSTM2C more slowly than the cardiomyocytes.

### Time course of GSTM2C uptake

**Fig 3:**
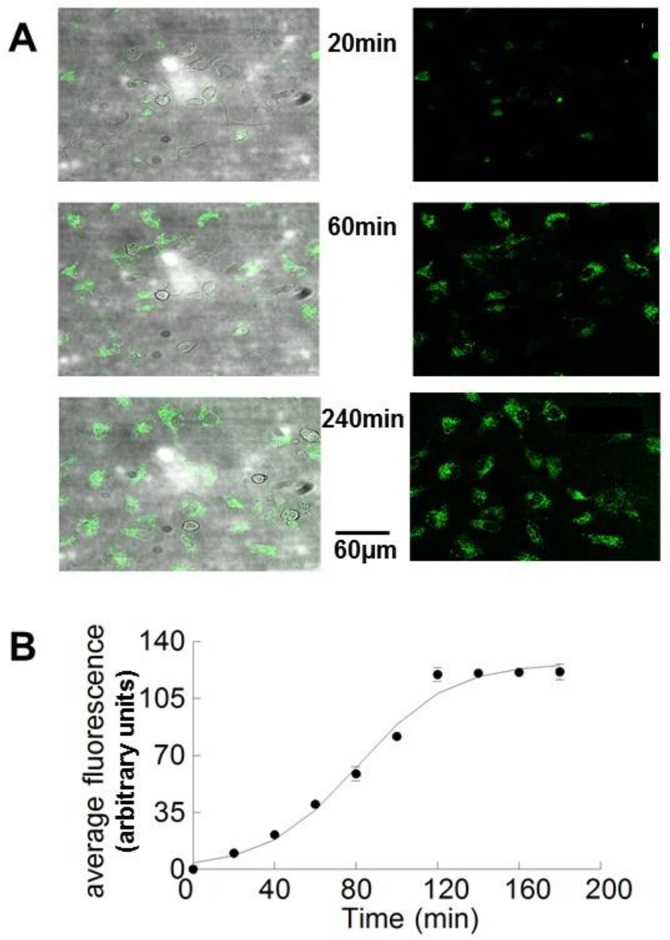
Time course of GSTM2C uptake into cardiomyocytes. Coverslips containing live cardiomyocytes were exposed to GSTM2C-OG for 3 to 4 h with images obtained every 20 min throughout the exposure. The field of view and detector settings were maintained throughout the experiment. **A**: Images after 20, 40 and 240 min exposure to 1 μM GSTM2C-OG. A transmission image is shown in the left and fluorescent image on the right. **B:** Mean fluorescence of cells. The average fluorescence within the field of view (in arbitrary units) ±1 SEM for the 10 experiments incubated with GSTM2C-OG for at least 180 min is plotted as a function of time (n = 10 for each time point). Standard error bars are not apparent when they fall within the dimensions of the symbol. The solid line shows a sigmoid curve fitted to the data.

To estimate the time course of GSTM2C uptake, myocytes were exposed to 1 μM GSTM2C-OG for 3–4 h and fluorescence images of the same field of view taken every 20 min. Examples of these lower magnification images taken after selected incubation periods of 20, 60 and 240 min exposure are shown in Fig 3A. It is notable that the fluorescence within the cells is considerably greater than that in the background solution indicating an active uptake of GSTM2C as reported previously [17]. Fluorescence is apparent within the cells after 20 min incubation, and increased up to a maximum between 60 and 240 min. Similar data was acquired at 20 min intervals up to 180 min in 10 experiments and the average data is shown in Fig 3B, with a sigmoid curve drawn through the data. A 2 h incubation period was considered optimal for cells to be maintained on the microscope stage at 37° C for subsequent contractility and Ca^2+^ transient experiments.

### Effect of GSTM2C on spontaneous contraction

**Fig 4:**
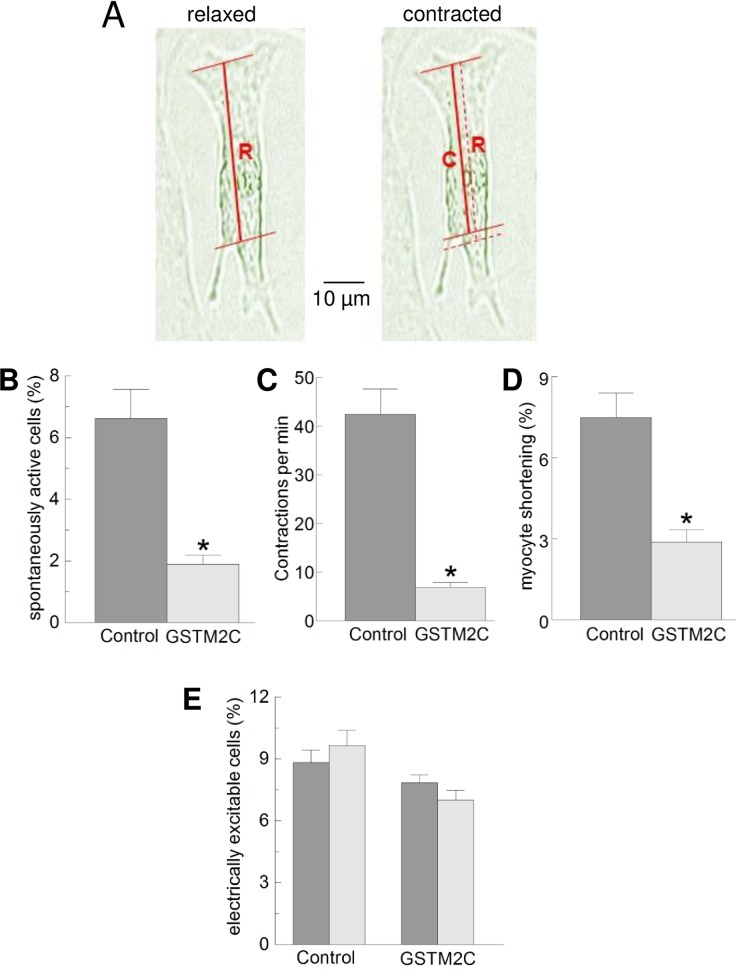
Contractile parameters of spontaneously contracting myocytes are reduced by GSTM2C, but the fraction of cells responding to electrical stimulation is unaffected. The contractile parameters of spontaneously contracting cardiomyocytes measured after 2 h incubation with either CDMEM media (control) or CDMEM media plus 1 μM GSTM2C. **A:** Transmission images of a myocyte in its maximally relaxed (left) and maximally shortened (right) state. The solid horizontal lines are through the same identifiable points in both the relaxed and contracted images. The thick red line is drawn between the identifiable points and indicate the measured lengths, **R** in the relaxed image and **C** in the contracted image. The thinner broken red line on the contracted image is the superimposed **R** length and is included to visualise the degree of shortening. Percentage shortening is given by ((**R**—**C**)/(**R**))*100. This method was also used in Fig 5C below. **B:** Percentage of spontaneously contracting cells (n = 20–30 fields of view). **C:** Frequency of spontaneous contractions (n = 30 myocytes). **D:** Myocyte shortening, with the most contracted (shortest) length expressed as a percentage of the relaxed length (n = 25 myocytes). All parameters were significantly reduced (P<0.001) after the cardiomyocytes were treated with GSTM2C, compared to exposure to the control solution. The data is presented as the mean ± SEM (measurements on myocytes from each of 5 separate cultures). **E:** The percentage of cells responding to external electrical stimulation measured before and then after exposure CDMEM media alone (control, n = 10 fields of view) or CDMEM plus 15 μM GSTM2C (n = 17 fields of view) for 2 h. Data was obtained from 5 different cultures. The field of view was not altered during the 2 h incubation. The data is presented as the mean ± SEM.

**Fig 5:**
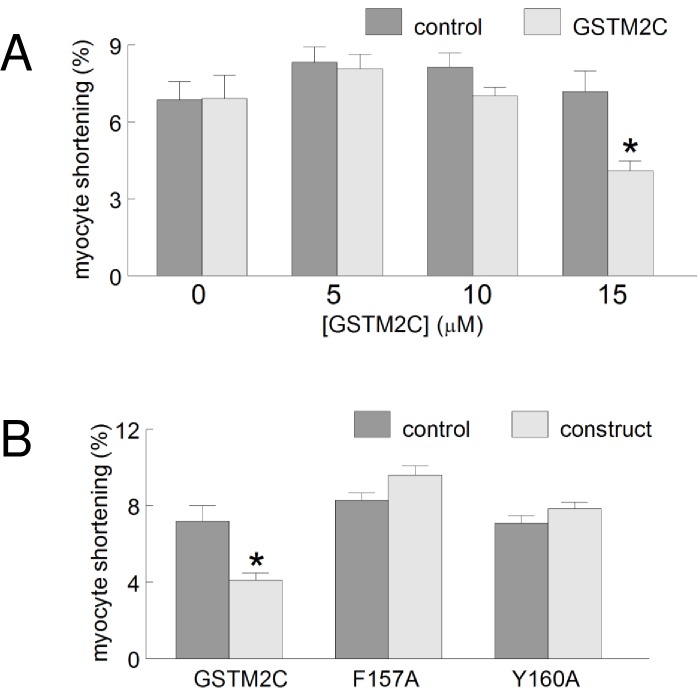
GSTM2C reduces field-stimulated myocyte shortening. **A:** The concentration -dependence of GSTM2C action on myocyte shortening. The percentage shortening was calculated for each myocyte before exposure to GSTM2C (control) and after (test) 2 h exposure to 5, 10 and 15 μM GSTM2C (n = 20–30 control myocytes and 20–30 myocytes after exposure to each concentration of GSTM2C, data from 5 different cultures). A significant reduction in shortening was observed only in the myocytes treated with 15 μM GSTM2C. **B:** A comparison of the activity of GSTM2 constructs on the percentage shortening. GSTM2C, n = 22 myocytes; F157A, n = 20 myocytes; Y160A, n = 27 myocytes. Data were obtained from 6 different cultures and are presented as mean ± SEM.

Both spontaneous and stimulated myocyte shortening was less than 10% of the measured length (Fig 4A, Fig 4D and Fig 5 below). This was of the same order of magnitude as that observed in freshly isolated adult myocytes and is much less than expected, likely due to myocyte adhesion to the coverslip [18]. Therefore cultured myocyte shortening is a very indirect measure of contractile properties and should be considered to be a qualitative rather than quantitative indication of the effect of GSTM2C on contraction. The fraction of myocytes demonstrating spontaneous contractions was significantly reduced (P<0.001) from 6.62 ± 0.92% in the control myocytes to 1.9 ± 0.28% after incubation with 1 μM GSTM2C, (Fig 4B). The contraction frequency was also significantly reduced, from 42.46 ± 5.18/min to 6.89 ± 0.92/min (P<0.001, Fig 4C) and the percentage shortening was significantly reduced from 7.48 ± 0.9% to 2.88 ± 0.45% (P<0.001 (Fig 4D). Spontaneous contraction frequency can depend on multiple factors. The rate of depolarization leading action potential generation in neonatal cardiomyocytes can be set by hyperpolarization-activated cyclic nucleotide-dependent (HCN) channels [27], and also by inward Na^+^ currents through the sodium/calcium exchange (NCX) activated when cytoplasmic [Ca^2+^] increases [28, 29]. Thus the reduced contraction frequency, as well as reduced shortening could be explained by the ability of GSTM2C to reduce the rate of Ca^2+^ release from isolated cardiac SR and inhibit RyR2 channels [12]. However the reduced frequency could also be attributed to effects on HCN channels, NCX and/or to action potential failure, if GCTM2C inhibited voltage-gated Ca^2+^ channels. To test the ability of myocytes to generate action potentials, we examined the effect of GSTM2C on their ability to contract in response to field stimulation.

### Effect of GSTM2C on myocyte response to field stimulation

For this experiment the concentration of GSTM2C was increased to 15 μM to ensure that both specific and non-specific actions of GSTM2C could be observed. We measured the fraction of myocytes responding to electrical stimulation, irrespective of the strength of the response, in order to explore possible effects of GSTM2C on the ability of the myocytes to generate action potentials. The number of myocytes that contracted in response to field stimulation was higher than the numbers of spontaneously contracting myocytes. The percentage of myocytes that responded to field stimulation was measured immediately before exposure to control CDMEM or GSTM2C-containing CDMEM and then measured again after 2 h exposure to the solutions. The field of view, temperature, stimulation frequency, duration and voltage were kept constant. The rationale was that, if action potential generation was affected by GSTM2C, the percentage of the myocytes that could be activated would be reduced after incubation in GSTM2C. However as shown in Fig 4E, the relative numbers of excitable myocytes were not significantly different between the pre- and post- incubation sessions, in either the absence or the presence of GSTM2C. Thus, it was concluded that action potential generation was not reduced by either the period of incubation or by exposure to GSTM2C. Although, with the present experiments, it remained possible that the GSTM2C altered action potential amplitude or duration, the reduced spontaneous contraction frequency with GSTM2C along with the reduced spontaneous shortening is consistent with the known inhibitory effects of GSTM2C on RyR2 [12]. RyR2 inhibition would lead to reduced Ca^2+^ leak through RyR2, lower cytoplasmic Ca^2+^ concentration, reduced NCX activity and Na^+^ influx and hence a reduction in the generation of spontaneous Ca^2+^ oscillations [12]. The effect of GSTM2C on shortening is also consistent with a reduced amount Ca^2+^ released from the SR in response to the spontaneous action potentials.

### Electrically stimulated contraction was reduced by 15 μM GSTM2C

As with spontaneous myocyte shortening, electrically stimulated shortening was reduced significantly after exposure to 15 μM of GSTM2C (Fig 5A and 5B). A trend towards a concentration-dependent reduction in contractility was observed between 5 μM, 10 μM to 15 μM GSTM2C, where the average shortening was 3.0%, 10.9% and 42.8% of control respectively (Fig 5A). Although the stimulation-evoked contractions were seen in the same number of myocytes before and after the 2 h incubation with 15 μM GSTM2C, the degree of shortening was significantly reduced (P<0.001). Such a result would be expected if action potential-induced Ca^2+^ release through RyR2 was reduced after incubation in GSTM2C as shown in adult cardiomyocytes [18]. In contrast to GSTM2C, neither 15 μM F157A nor 15 μM Y160A altered field-stimulated shortening (Fig 5B), consistent with the lack of an effect of these constructs on single channel activity or on Ca^2+^ release from cardiac SR [12].

### Effect of GSTM2C constructs on electrically evoked Ca^2+^ transients

**Fig 6:**
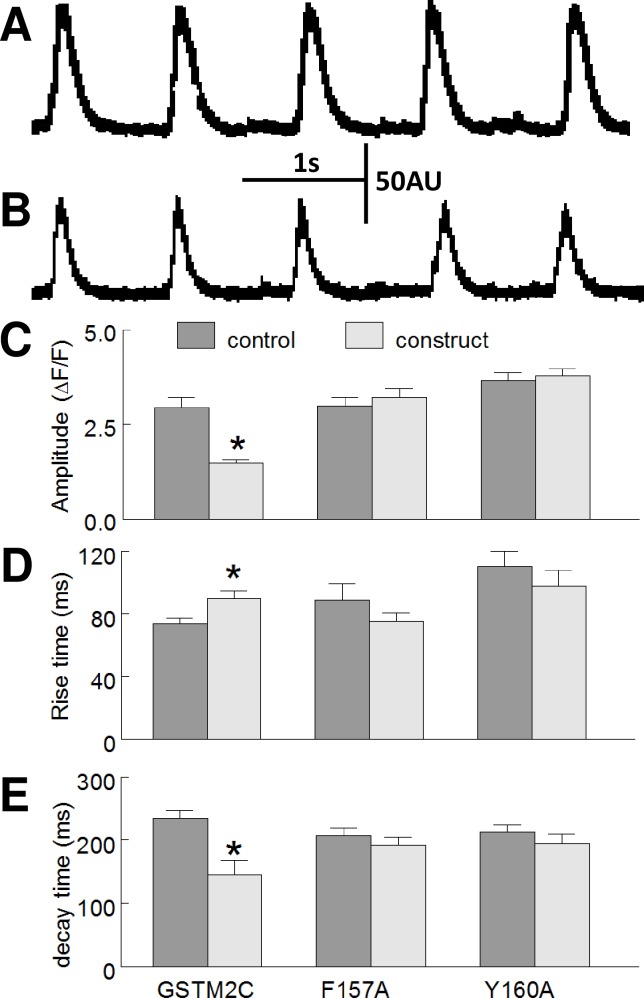
GSTM2C alters Ca^2+^ cycling in field stimulated myocytes. **A and B:** A train of 5 Ca^2+^ transients evoked at 1 Hz in one myocyte before (**A**) and after (**B**) exposure to 15 μM GSTM2C in arbitrary units (AU). **C-E:** The effect of 15 μM GSTM2C, F157A and Y160A on the amplitude of the Ca^2+^ transient (ΔF/F0) (**C**); rise time (from 10 to 90% of the peak amplitude) in ms (**D**); and the decay time (from peak to 50%) in ms. There are significant effects on each of the parameters with GSTM2C, but not with either mutant. The data are presented as the mean ± SEM (n = 22 myocytes for GSTM2C; n = 25 myocytes for F157A; n = 23 myocytes for Y160A; from 5 to 6 separate cultures).

The peak amplitude, rise time and decay time were measured for each Ca^2+^ transient in the train of five pulses (Fig 6A and 6B). Control Ca^2+^ transients were obtained from untreated myocytes and test Ca^2+^ transients obtained after 2 h exposure to 15 μM GSTM2C, F157A or Y160A. The amplitude of the Ca^2+^ transient in the GSTM2C- treated cells was significantly less than amplitude of the Ca^2+^ transient in the untreated control myocytes (P<0.001) (Fig 6C). The rise time, i.e. the time taken for cytosolic Ca^2+^ to increase from 10 to 90%, increased significantly in the GSTM2C-treated cells (Fig 6D) suggesting a reduced rate of Ca^2+^ release from the SR. In addition, there was a significant reduction in the decay time, i.e. the time taken for 50% of Ca^2+^ in the sarcoplasm to be removed in the presence of GSTM2C (Fig 6E). The nett rate of Ca^2+^ uptake is equal to the rate of influx into the SR via SERCA minus the rate of Ca^2+^ efflux (leak) from the SR through RyR2. Therefore, this result is again consistent with a reduced Ca^2+^ leak from the SR during the Ca^2+^ uptake phase of Ca^2+^ cycling in the presence of GSTM2C. The slower rate of Ca^2+^ release and the faster removal of Ca^2+^ most likely combined to produce the reduced amplitude of the Ca^2+^ transient. Neither the amplitude, decay time nor rise time were significantly altered by the F157A or Y160 mutants (Fig 6C–6E).

## Discussion

In this study we provide novel evidence that GSTM2C can enter neonatal cardiomyocytes and can profoundly alter the properties of the cells, reducing spontaneous activity and the ability of the cells to shorten, consistent with an inhibition of Ca^2+^ release from the SR during spontaneous contractions and contraction in response to electrical field stimulation. The results are consistent with the effects of GSTM2C on intact freshly isolated adult cardiomyocytes [18] and are also consistent with a specific inhibitory effect of GSTM2C on Ca^2+^ release from isolated cardiac SR vesicles and on the open probability of cardiac RyR2 channels [11, 12]. Although we cannot exclude the possibility that GSTM2C interacts with other components of the Ca^2+^ cycling machinery, the results are consistent with the well-documented inhibitory action of the compounds on the cardiac RyR2 channel.

### Ca^2+^ cycling in neonatal cardiomyocytes

The Ca^2+^ cycling machinery in cultured neonatal cardiomyocytes differs from that in freshly dissociated adult cardiomyocytes in several ways [30], including the presence of IP3 sensitive Ca^2+^ stores that contribute to Ca^2+^ cycling [21]. However the spontaneous activity of both the neonatal myocytes and adult cardiomyoctes reflects the activity of ion channels and NCX in the surface membrane. There is disparity in the literature in regard to spontaneous beating of neonatal myocytes, with some studies suggesting that the activity depends only on pacemaker currents though HCN2 and HCN4 channels [27], while other studies suggest that spontaneous oscillations in Ca^2+^ release from the SR in fact drive the electrical activity and contractions, via activation of depolarizing Na^+^ current as NCX imports 3 Na^+^ ions for each Ca^2+^ extruded [29]. Indeed both mechanisms can co-exist and both contribute to spontaneous beating [28]. The expression of RyR2 is less than in adult cardiomyocytes and thus the contribution of ryanodine sensitive Ca^2+^ stores to Ca^2+^ transients is likely to be less than in adult cells [21]. Never-the-less, blocking RyR2 channels in neonatal cardiomyocytes by either depleting ryanodine -sensitive stores with ryanodine or caffeine, or by blocking RyR2 with tetracaine, profoundly reduces both IP3 and caffeine-induced Ca^2+^ release [31]. The results presented in this manuscript show that the effects of GSTM2C on neonatal cardiac myocytes are consistent with the known actions of GSTM2C on RyR2 channel activity. The significance of the question is not only basic, but also lies in the potential use of GSTM2C or its derivatives as drugs to reduce excess Ca^2+^ release in juvenile cardiomyopathies with arrhythmia attributed to high diastolic Ca^2+^ levels due to excess Ca^2+^ leak through RyR2.

### GSTM2C translocation into cardiomyocytes

The mode of entry of GSTM2C into cardiomyocytes was not investigated here, but as shown previously [20] GSTM2C is transported into other cells by endocytosis and it is likely that the same mode of entry is employed in neonatal ventricular myocytes. The dependence on an active process was also indicated here by the fact that the fluorescence in the myocytes was considerably greater than that in the bathing solution (Fig 3A). There is evidence that the mutations in the helix 6 region of GSTM2C may destabilize the structure of the molecule and expose the hydrophobic helix 6 region and enhance the ability of the mutants to enter cells [26]. Indeed the two destabilizing mutations, F157A and Y160A do enhance the uptake of the protein into L-929 cells and into adult mouse cardiomyocytes [18, 20]. The enhanced cellular uptake of the two GSTM2C mutants indicated that the capacity of the GSTM2C to enter cells is inversely related to protein stability [20]. Despite the enhanced cellular uptake of the mutants [20], the fact that they did not significantly alter Ca^2+^ transients and contractility suggests that residues F157 and Y160 are involved in the direct interaction between GSTM2C and RyR2.

### GSTM2C-induced inhibition spontaneous contraction frequency

As in adult myocytes [32], spontaneous contractions in neonatal cardiomyocytes depend on action potentials triggered when depolarization, generated by an inward current across the surface membrane, reaches action potential threshold. Such inward currents can be generated by surface membrane HCN2 and HCN4 channels [27] and/or follow activation of the NCX by increases in cytoplasmic [Ca^2+^] following release from IP3 and/or ryanodine sensitive stores [28, 31]. The effect of GSTM2C on the number of spontaneously active cells and on the frequency of spontaneous contractility most likely depends on an effect of the drug on Ca^2+^ leak through RyR2, since GSTM2C is a specific inhibitor of RyR2 channels [11, 12, 16]. Indeed a BLAST alignment indicates that the specific GSTM2C binding sequence in the RyR2 D3 region [16] is not present in rat or human HCN2. There is a region of alignment in HCN4, but only one identical residue was found to lie within the GSTM2C-binding region. This single residue is unlikely to support GSTM2C binding as GSTM2C does not bind to the D3 region of RyR1, despite four identical residues in the GSTM2C-binding region.

Arrhythmia in neonatal cardiomyocytes can be generated in a similar manner to spontaneous activity in adult cardiomyocytes in that delayed after depolarisations (DADs) are generated when the NCX is activated during diastole by an increase in cytoplasmic Ca^2+^ as a result of excess Ca^2+^ leak through RyR2. Thus the ability of GSTM2C to reduce the spontaneous activity in neonatal cardiomyocytes suggests that the drug may also be effective in reducing spontaneous activity leading arrhythmia in the neonatal heart.

### Inhibition of electrically-evoked Ca^2+^ transients and contractility

The effects of GSTM2C on contractility and Ca^2+^ transients in electrically stimulated myocytes was examined in order to avoid the individual variability seen in spontaneously active cells and to simulate regular pacing of myocytes in the intact heart. Following exposure to GSTM2C the degree of myocyte shortening was reduced presumably as a result of the smaller Ca^2+^ transient. That the rate of rise of the Ca^2+^ transient was slower after GSTM2C treatment can be explained by the known actions of GSTM2C in specifically blocking RyR2 and thus reducing the rate of Ca^2+^ release in response to the action potential. On the other hand, the decay of the Ca^2+^ transient indicates the rate of removal of Ca^2+^ from the cytoplasm and is effectively set by the rate of Ca^2+^ extrusion and uptake into the SR, minus any Ca^2+^ leak back into the cytoplasm through RyR2. This inhibition of RyR2 leak would increase the rate of Ca^2+^ uptake as we observed as back flux into cytoplasm would be reduced as mentioned in the Results. The slower release rate combined with the faster removal rate would account for the reduced amplitude of the Ca^2+^ transient.

A smaller Ca^2+^ transient amplitude could also be caused by a reduced Ca^2+^ load in the SR with GSTM2C. A reduced Ca^2+^ load occurs with increased Ca^2+^ leak through RyR2 or with reduced SERCA2 (sarcoplasmic/endoplasmic reticulum Ca^2+^ ATPase) activity. However we know that RyR2 activity is inhibited by GSTM2C [12], but that SERCA2 activity is unaffected by GSTM2C [18] in adult freshly isolated myocytes, so that the net effect that would be an increase in store load. As the isoforms of the proteins (RyR2 and SERCA2a) are the same in adult and neonatal cardiomyocytes it seems unlikely that Ca^2+^ load would be reduced in the neonatal myocytes. Indeed the enhanced uptake rate during Ca^2+^ removal from the cytoplasm during the Ca^2+^ transients is consistent with inhibition of RyR2 in both preparations. Further evidence that Ca^2+^ load in the SR is not reduced, and that there was no greater than normal Ca^2+^ extrusion across the surface membrane is provided by the fact that the amplitude of the Ca^2+^ transient did not decline during the AP train in the presence of GSTM2C any more than it did under control conditions. If Ca^2+^ uptake was reduced, the Ca^2+^ load in the SR would decline during the stimulus train and result in a rapid decline in the amplitude of the Ca^2+^ transients.

The concentration dependence of the effects of GSTM2C, and the lack of an effect of F157A and Y160A, was similar on RyR2 in isolated cardiac SR and on myocyte contractility and Ca^2+^ transients in intact cells. This suggests that the effects of GSTM2C on myocytes is at least partly a result of GSTM2C interactions with RyR2. We show that there is little effect on the ability of field stimulation to evoke contractions, indicating that the action potential generation mechanism was not altered by GSTM2C. We did not specifically examine the effects of the constructs on other factors such as SERCA or NCX activity. However the fact that the specific inhibition of the cardiac RyR2 depends on GSTM2C binding to a cardiac RyR2-specific sequence in the divergent region 3 of RyR2 [16] suggests that the compound would be unlikely to interact with other proteins that do not contain this specific sequence.

### The potential of GSTM2C as a therapeutic agent

As mentioned above, the ability of GSTM2C to reduce spontaneous contractions in the neonatal cardiomyocytes suggests that it may be effective in blocking arrhythmia due to NCX activation by high cytosolic Ca^2+^ during diastole in both neonatal and adult heart. It is possible that a reduction in the amplitude of the Ca^2+^ transient and fiber shortening in both neonatal (reported here) and in adult myocytes [18], might reduce the therapeutic benefit of the compound. However this remains to be fully investigated. It is notable that 5 μM GSTM2C produces significant depression of RyR2 channels [12] and we show here that it enters myocytes at an external concentration of only 1 μM, but is likely concentrated in endosomes [17]. The fact that GSTM2C has effects consistent with RyR2 inhibition indicates that it escapes from the endosomes into the cytoplasm, although the precise cytoplasmic concentration is not known, it is likely to be at least 5 μM. Importantly GSTM2C suppressed spontaneous contractions, indicative of efficacy in suppressing spontaneous arrhythmogenic activity, at an extracellular concentration of only 1 μM. In marked contrast contractility in externally paced myocytes, reflecting contractility in self-paced myocytes in the intact heart, was not significantly altered till the extracellular concentration was increased to 15 μM. Therefore it seems likely that there is a window of concentrations where the compound may suppress spontaneous activity without altering coordinated paced activity.

In addition, we predict that GSTM2C may not have the same effects on the Ca^2+^ transient and contraction in myocytes electrically stimulated with a brief train of pulses and in continuous self-paced activity in intact tissue. It is likely that altered systolic Ca^2+^ release may not be maintained in continually active cells where an auto-correcting mechanism can maintain optimal systolic Ca^2+^ transient amplitude [33]. In contrast there is no auto-correction during diastole where excess Ca^2+^ leak from the SR can cause the DADS that lead to arrhythmia. We did not see Ca^2+^ transient auto-correction in acutely isolated adult myocytes [18], where the responses to the brief stimulation protocol were dominated by the decay of post-rest potentiation, or in the cultured neonatal cardiomyocytes reported here.

## Conclusions

We show here that GSTM2C is translocated into the cytoplasm of neonatal cardiomyocytes and is effective in reducing spontaneous contractility and that its actions on field stimulated Ca^2+^ release and contraction is very similar to that reported for adult cardiomyocytes [18]. All results are consistent with a highly specific action of GSTM2C on the cardiac RyR2 due to the drug’s binding to an amino acid sequence found only in the cardiac isoform of the RyR [16]. The results further highlight the potential of GSTM2C for therapeutic use for genetic and acquired arrhythmia that depends on excess Ca^2+^ release from the SR through RyR2 in both juvenile and adult conditions. The study shows for the first time that the drug is equally effective in cultured adult and neonatal cardiomyocytes.

## References

[1] Hayes JD, Pulford DJ. The glutathione S-transferase supergene family: regulation of GST and the contribution of the isoenzymes to cancer chemoprotection and drug resistance. Crit Rev Biochem Mol Biol. 1995;30(6):445–600. 10.3109/10409239509083491 . 8770536

[2] Adler V, Yin Z, Fuchs SY, Benezra M, Rosario L, Tew KD, et al Regulation of JNK signaling by GSTp. EMBO J. 1999;18(5):1321–34. 10.1093/emboj/18.5.1321 10064598 PMC1171222

[3] Cho SG, Lee YH, Park HS, Ryoo K, Kang KW, Park J, et al Glutathione S-transferase mu modulates the stress-activated signals by suppressing apoptosis signal-regulating kinase 1. J Biol Chem. 2001;276(16):12749–55. 10.1074/jbc.M005561200 . 11278289

[4] Wu Y, Fan Y, Xue B, Luo L, Shen J, Zhang S, et al Human glutathione S-transferase P1-1 interacts with TRAF2 and regulates TRAF2-ASK1 signals. Oncogene. 2006;25(42):5787–800. 10.1038/sj.onc.1209576 . 16636664

[5] Johansson AS, Mannervik B. Human glutathione transferase A3-3, a highly efficient catalyst of double-bond isomerization in the biosynthetic pathway of steroid hormones. J Biol Chem. 2001;276(35):33061–5. 10.1074/jbc.M104539200 . 11418619

[6] Blackburn AC, Woollatt E, Sutherland GR, Board PG. Characterization and chromosome location of the gene GSTZ1 encoding the human Zeta class glutathione transferase and maleylacetoacetate isomerase. Cytogenet Cell Genet. 1998;83(1–2):109–14. 15145. . 9925947 10.1159/000015145

[7] Fernandez-Canon JM, Hejna J, Reifsteck C, Olson S, Grompe M. Gene structure, chromosomal location, and expression pattern of maleylacetoacetate isomerase. Genomics. 1999;58(3):263–9. 10.1006/geno.1999.5832 . 10373324

[8] Laliberte RE, Perregaux DG, Hoth LR, Rosner PJ, Jordan CK, Peese KM, et al Glutathione s-transferase omega 1–1 is a target of cytokine release inhibitory drugs and may be responsible for their effect on interleukin-1beta posttranslational processing. J Biol Chem. 2003;278(19):16567–78. 10.1074/jbc.M211596200 . 12624100

[9] Abdellatif Y, Liu D, Gallant EM, Gage PW, Board PG, Dulhunty AF. The Mu class glutathione transferase is abundant in striated muscle and is an isoform-specific regulator of ryanodine receptor calcium channels. Cell Calcium. 2007;41(5):429–40. 10.1016/j.ceca.2006.08.004 . 17023043

[10] Board PG, Suzuki T, Shaw DC. Human muscle glutathione S-transferase (GST-4) shows close homology to human liver GST-1. Biochim Biophys Acta. 1988;953(3):214–7. . 3281712 10.1016/0167-4838(88)90027-1

[11] Liu D, Hewawasam R, Pace SM, Gallant EM, Casarotto MG, Dulhunty AF, et al Dissection of the inhibition of cardiac ryanodine receptors by human glutathione transferase GSTM2-2. Biochem Pharmacol. 2009;77(7):1181–93. 10.1016/j.bcp.2008.12.024 . 19168034

[12] Hewawasam R, Liu D, Casarotto MG, Dulhunty AF, Board PG. The structure of the C-terminal helical bundle in glutathione transferase M2-2 determines its ability to inhibit the cardiac ryanodine receptor. Biochem Pharmacol. 2010;80(3):381–8. 10.1016/j.bcp.2010.04.019 . 20417188

[13] Dulhunty AF, Haarmann CS, Green D, Laver DR, Board PG, Casarotto MG. Interactions between dihydropyridine receptors and ryanodine receptors in striated muscle. Prog Biophys Mol Biol. 2002;79(1–3):45–75. . 12225776 10.1016/s0079-6107(02)00013-5

[14] Meissner G. Regulation of mammalian ryanodine receptors. Front Biosci. 2002;7:d2072–80. . 12438018 10.2741/A899

[15] Aceto A, Dragani B, Melino S, Allocati N, Masulli M, Di Ilio C, et al Identification of an N-capping box that affects the alpha 6-helix propensity in glutathione S-transferase superfamily proteins: a role for an invariant aspartic residue. Biochem J. 1997;322 (Pt 1):229–34. 9078266 10.1042/bj3220229 PMC1218181

[16] Liu D, Hewawasam R, Karunasekara Y, Casarotto MG, Dulhunty AF, Board PG. The inhibitory glutathione transferase M2-2 binding site is located in divergent region 3 of the cardiac ryanodine receptor. Biochem Pharmacol. 2012;83(11):1523–9. 10.1016/j.bcp.2012.02.020 . 22406107

[17] Morris MJ, Craig SJ, Sutherland TM, Board PG, Casarotto MG. Transport of glutathione transferase-fold structured proteins into living cells. Biochim Biophys Acta. 2009;1788(3):676–85. 10.1016/j.bbamem.2008.10.018 . 19038230

[18] Samarasinghe K, Liu D, Tummala P, Cappello J, Pace SM, Arnolda L, et al Glutathione transferase M2 variants inhibit ryanodine receptor function in adult mouse cardiomyocytes. Biochem Pharmacol. 2015;97(3):269–80. 10.1016/j.bcp.2015.08.004 . 26256076

[19] Raghunathan S, Chandross RJ, Kretsinger RH, Allison TJ, Penington CJ, Rule GS. Crystal structure of human class mu glutathione transferase GSTM2-2. Effects of lattice packing on conformational heterogeneity. J Mol Biol. 1994;238(5):815–32. 10.1006/jmbi.1994.1336 . 8182750

[20] Morris MJ, Liu D, Weaver LM, Board PG, Casarotto MG. A structural basis for cellular uptake of GST-fold proteins. PLoS One. 2011;6(3):e17864 10.1371/journal.pone.0017864 21455499 PMC3063774

[21] Jaconi M, Bony C, Richards SM, Terzic A, Arnaudeau S, Vassort G, et al Inositol 1,4,5-trisphosphate directs Ca(2+) flow between mitochondria and the Endoplasmic/Sarcoplasmic reticulum: a role in regulating cardiac autonomic Ca(2+) spiking. Mol Biol Cell. 2000;11(5):1845–58. 10793156 10.1091/mbc.11.5.1845 PMC14888

[22] Gomez JP, Potreau D, Raymond G. Intracellular calcium transients from newborn rat cardiomyocytes in primary culture. Cell Calcium. 1994;15(4):265–75. . 8055543 10.1016/0143-4160(94)90066-3

[23] Baker RT, Catanzariti AM, Karunasekara Y, Soboleva TA, Sharwood R, Whitney S, et al Using deubiquitylating enzymes as research tools. Methods Enzymol. 2005;398:540–54. 10.1016/S0076-6879(05)98044-0 . 16275357

[24] Field AC, Hill C, Lamb GD. Asymmetric charge movement and calcium currents in ventricular myocytes of neonatal rat. J Physiol. 1988;406:277–97. 2855436 10.1113/jphysiol.1988.sp017380 PMC1191099

[25] Louch WE, Sheehan KA, Wolska BM. Methods in cardiomyocyte isolation, culture, and gene transfer. J Mol Cell Cardiol. 2011;51(3):288–98. 10.1016/j.yjmcc.2011.06.012 21723873 PMC3164875

[26] Komiyama M, Maruyama K, Shimada Y. Assembly of connectin (titin) in relation to myosin and alpha-actinin in cultured cardiac myocytes. J Muscle Res Cell Motil. 1990;11(5):419–28. . 2266168 10.1007/BF01739762

[27] Er F, Larbig R, Ludwig A, Biel M, Hofmann F, Beuckelmann DJ, et al Dominant-negative suppression of HCN channels markedly reduces the native pacemaker current I(f) and undermines spontaneous beating of neonatal cardiomyocytes. Circulation. 2003;107(3):485–9. . 12551875 10.1161/01.cir.0000045672.32920.cb

[28] Rapila R, Korhonen T, Tavi P. Excitation-contraction coupling of the mouse embryonic cardiomyocyte. J Gen Physiol. 2008;132(4):397–405. 10.1085/jgp.200809960 18794377 PMC2553387

[29] Sasse P, Zhang J, Cleemann L, Morad M, Hescheler J, Fleischmann BK. Intracellular Ca2+ oscillations, a potential pacemaking mechanism in early embryonic heart cells. J Gen Physiol. 2007;130(2):133–44. 10.1085/jgp.200609575 17664344 PMC2151640

[30] Korhonen T, Hanninen SL, Tavi P. Model of excitation-contraction coupling of rat neonatal ventricular myocytes. Biophys J. 2009;96(3):1189–209. 10.1016/j.bpj.2008.10.026 19186154 PMC2716686

[31] Janowski E, Berrios M, Cleemann L, Morad M. Developmental aspects of cardiac Ca(2+) signaling: interplay between RyR- and IP(3)R-gated Ca(2+) stores. Am J Physiol Heart Circ Physiol. 2010;298(6):H1939–50. 10.1152/ajpheart.00607.2009 20304819 PMC2886654

[32] Maltsev VA, Vinogradova TM, Lakatta EG. The emergence of a general theory of the initiation and strength of the heartbeat. J Pharmacol Sci. 2006;100(5):338–69. . 16799255 10.1254/jphs.cr0060018

[33] Trafford AW, Sibbring GC, Diaz ME, Eisner DA. The effects of low concentrations of caffeine on spontaneous Ca release in isolated rat ventricular myocytes. Cell Calcium. 2000;28(4):269–76. 10.1054/ceca.2000.0156 . 11032782

